# Endothelial-Derived Interleukin-1α Activates Innate Immunity by Promoting the Bactericidal Activity of Transendothelial Neutrophils

**DOI:** 10.3389/fcell.2020.00590

**Published:** 2020-07-07

**Authors:** Xiaoye Liu, Hui Zhang, Shangwen He, Xiang Mu, Ge Hu, Hong Dong

**Affiliations:** ^1^Beijing Traditional Chinese Veterinary Engineering Center and Beijing Key Laboratory of Traditional Chinese Veterinary Medicine, Beijing University of Agriculture, Beijing, China; ^2^Department of Mechanics and Engineering Science, College of Engineering, Academy for Advanced Interdisciplinary Studies, and Beijing Advanced Innovation Center for Engineering Science and Emerging Technology, College of Engineering, Peking University, Beijing, China; ^3^Beijing Advanced Innovation Center for Food Nutrition and Human Health, College of Veterinary Medicine, China Agricultural University, Beijing, China

**Keywords:** endothelial-derived interleukin-1α, transendothelial neutrophils, lipopolysaccharide, *Escherichia coli* infection, iTRAQ

## Abstract

Migration of neutrophils across endothelial barriers to capture and eliminate bacteria is served as the first line of innate immunity. Bacterial virulence factors damage endothelium to produce inflammatory cytokines interacts with neutrophils. However, the mechanisms that behind endothelial-neutrophil interaction impact on the bactericidal activity remain unclear. Therefore, we aimed to find the target proteins on endothelial cells that triggered the bactericidal activity of transendothelial neutrophils. Herein, we built the infected models on rats and endothelial-neutrophil co-cultural system (Transwell) and discovered that endothelial-derived IL-1α promoted the survival of rats under *Escherichia coli* infection and enhanced the bactericidal activity of transendothelial neutrophils *in vivo* and *in vitro*. Results further showed that IL-1α was inhibited by lipopolysaccharide (LPS) in the endothelial-neutrophil interaction. We found that LPS mainly damaged cell membrane and induced cell necrosis to interrupt neutrophil migration from endothelial barrier. Thus, we used the isobaric tags for relative and absolute quantification (iTRAQ) method to identify different proteins of endothelial cells. Results showed that IL-1α targeted cellular plasma membrane, endoplasmic reticulum and mitochondrial envelope and triggered eleven common proteins to persistently regulate. During the early phase, IL-1α triggered the upregulation of cell adhesion molecules (CAMs) to promote neutrophil adhesion, while oxidative phosphorylation was involved in long time regulation to induce transmigration of neutrophils against bacteria. Our results highlight the critical mechanism of endothelial-derived IL-1α on promoting bactericidal activity of transendothelial neutrophils and the findings of IL-1α triggered proteins provide the potentially important targets on the regulation of innate immunity.

## Introduction

Endothelial cells are the inner cell lines connected with immune cells and epithelium (Rohlenova et al., 2018). One kind of immune cells, neutrophils, must across endothelial cells to reach the infected sites against pathogenic infection (Papayannopoulos, 2018). In turn, bacteria employ their virulence factors to hijack endothelial cells and induce inflammatory cytokine release as the major strategy to break through epithelium barrier and inhibit innate immune system (Liu et al., 2017; Yuan et al., 2018). For instance, the lipopolysaccharide (LPS) secreted from *Escherichia coli* (*E. coli*) impacts the release of inflammatory mediators to regulate the progress of infection by leading immune cell damage (Li et al., 2016; Presicce et al., 2020). It worth to note that the inflammatory cytokine, interleukin-1α (IL-1α) can induce neutrophil extracellular traps (NETs) to activity endothelial cell (Folco et al., 2018). As similar as our previous research illustrated that endothelial IL-1α enhanced the bacterial killing of transendothelial neutrophils (Liu et al., 2016). In addition, IL-1α is primarily associated with inflammatory during the pathogenesis induced by bacterial infection (Dinarello, 2011; Menghini et al., 2019). However, the mechanisms of how endothelial-derived IL-1α regulate the killing ability of transendothelial neutrophils remain unknown. Thus, we hypothesized that endothelial IL-1α modulated endothelial cells to impact bacterial killing of transendothelial neutrophils.

In this work, we aimed to investigate how endothelial-derived IL-1α impacted the bactericidal activity of transendothelial neutrophils during endothelial-neutrophil interaction though two *E. coli* infected models of rats and endothelial-neutrophil co-cultural system (Transwell). Further, we intended to find the regulated difference proteins on endothelial cells that triggered by IL-1α via using iTRAQ-based quantitative proteomics.

## Materials and Methods

### Animals

Rats (1-day rats and 1–2-month rats) were purchased from academy of military medical sciences, Beijing, China (Certificate Number: SCXK-PLA 2012-0004). One day rats were obtained to isolate primary RIMVECs and 1–2-months rats were used for the rat infection.

### Ethics Statement

The experimental protocols involving rats were gained an approval by the Institutional Animal Care and Use Committee of the Academy of Military Medical Sciences (Beijing, China; approval no. SYXK2014-0002).

### Rat Infection

Rats (1–2 month, about 500 g, 10 rats per group) were infected with 10^9^ colony-forming units (CFUs) of *E. coli* (serotype O55:B5) orally. To simulate the situation of stress-induced LPS accumulation. We set up the group of additional LPS by adding 1 μg/g of LPS (from *E. coli* serotype O55:B5, Sigma-Aldrich) mixed with *E. coli* suspension. After 24 h infection, IL-1α, IL-1β, IL-6, intercellular adhesion molecule-1 (ICAM-1) and Tumor Necrosis Factor (TNF-α) from rat serums were detected by the ELISA kits (BD Biosciences) according to the instructions. For further investigating the survival of *E. coli* infected rats, simultaneous addition of IL-1α (rat recombinant, Sigma-Aldrich) with 10 ng/g for each infected group. Then the ratios of rat survival were recorded. Lastly the *E. coli* that survived in rat colons were detected by the colony count technique (colony-forming units, CFUs).

### Primary Endothelial Cell Culture

Primary rat intestinal mucosal microvascular endothelial cells (RIMVECs) were separated from the colons of 1 day-rats and then cultured in complete Dulbecco’s modified eagle medium (DMEM, Gibco) containing 2 mM L-glutamic acid, 50 mg/l gentamycin, 100 U/mL penicillin/streptomycin and 20% heat-inactivated fetal bovine serum (FBS, Gibco). The identification of RIMVECs was obtained as previous protocol (Liu et al., 2016).

### Isolation of Blood Neutrophils

Rat fresh neutrophils were isolated from heparinized whole blood of healthy rats by gradient centrifugation assay using Percoll reagent (GE Healthcare) as previous published methods (Liu et al., 2016). Then neutrophils were washed with HBSS and preserved in RPMI-1640 medium (Gibco) for later use after counting and viability assessment.

### Detecting the Damage of LPS on RIMVECs

RIMVECs (1 × 10^4^ cells/well) were seeded in a 96-well plate and treated with a final concentrations of 1 μg/mL LPS for different time points (0.5, 1, 2, 4, 8, 12, and 24 h) at 37°C in a 5% CO_2_ atmosphere. After treatment, the cytotoxicity of RIMVECs was detected by 10 μL of WST-1 reagents (Roche). After 1 h incubation at 37°C, the absorbance was detected by a fluorescence microplate reader (Life Science & Technology) at wavelength of 450 nm. The percentage of RIMVECs survival was calculated based on the ratio of absorbance compared to DMEM treated group. After RIMVECs treated with LPS, then cells were washed with PBS and incubated with PI (5 μg/mL, Sigma-Aldrich) for 30 min. The PI positive cells presented the membrane damaged cells and fluorescence intensity of PI was immediately detected with excitation wavelength at 535 nm and emission wavelength at 615 nm.

### Flow Cytometry

To record the proportion of necrosis and apoptosis on RIMVECs leaded by LPS, we used an Annexin-V-FITC (Annexin-V-fluorescein isothiocyanate) and propidium iodide (PI) double staining kit (B&D system) to track the cytotoxicity of LPS. Annexin-V was employed to label membrane phosphatidylserine on the surface of early apoptotic cells, which displayed green fluorescence due to FITC. PI was used to sort the necrotic cells by further binding to cellular DNA and showing red fluorescence. Detection and analysis of necrosis were used BD FACSAria^TM^ flow cytometry and FACSDiva software (BD Biosciences) based as our previous publish method (Liu et al., 2017).

### Infection of the Endothelial-Neutrophil Interaction

**FIGURE 1:**
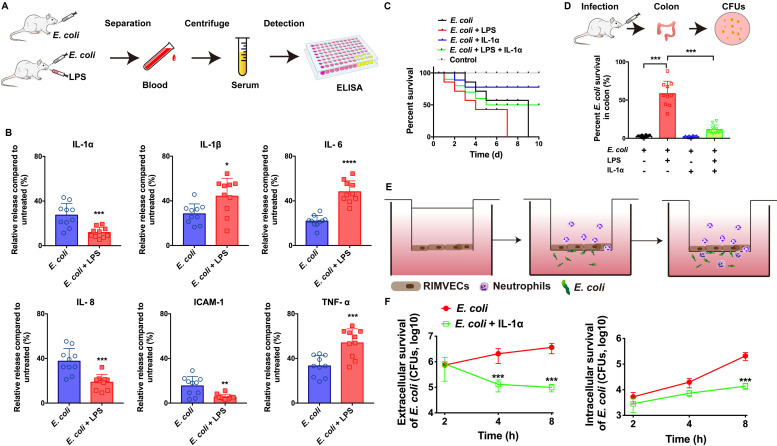
IL-1α facilitated the bacterial killing on the *in vivo* and *in vitro* model of *E. coli* infection. **(A)** *E. coli* infection *in vivo* rat model workflow. **(B)** IL-1α, IL-1β, IL-6, IL-8, ICAM-1, and TNF-α were detected by ELISA. Rats were infected by *E. coli* (10^9^ CFU/g) with or without LPS (1 μg/mL) treatment. After 24 h infection, the serum of every rats including both dead or live rats were collected to detect the inflammation-associated cytokines by ELISA. **(C)** IL-1α increase the survival of LPS-induced rat death. A total of 10^9^ CFU/g *E. coli* were infected with rats for 24 h. LPS (1 μg/g) or IL-1α (10 ng/g) were treated with the infected rats. The ratios of rat survival were determined from 10 days’ treatment. Gray line showed the percent survival of uninfected rats, red line represented the *E. coli* infection, blue line was the LPS treatment of *E. coli* infection, green line represented LPS plus IL-1α treated with *E. coli* infected rats. **(D)** IL-1α decreased the bacterial load in rat colon. The number of bacteria was counted by the colony count technique (CFU). Percentage of *E. coli* were compared to untreated group. Data are shown as means ± SD (*P < 0.05; **P < 0.01; ***P < 0.001, *n* = 10). **(E)** Scheme of co-culture system for RIMVECs-neutrophils interactions *in vitro*. RIMVECs were seeded on upper chambers for 24 h to form monolayer. Then neutrophils were added in the upper chambers. *E. coli* infected **(F)** IL-1α increased the transendothelial neutrophils killing. *E. coli* infected in bottom chambers treated for different time points (2, 4, and 8 h) and IL-1α (10 ng per well) was also treated in the upper chambers. Both the extracellular and intracellular bacteria in neutrophils (5 × 10^8^ cells) were used to count the *E. coli* CFUs. The red lines represented *E. coli* infection and green lines showed the IL-1α treatment of *E. coli* infection. Values represent the mean ± SD (***P < 0.001, *n* = 6).

RIMVECs (1 × 10^4^ cells/well) were seeded onto the 5.0 μm pore size polycarbonate resin transwell membranes to reach confluence and form a monolayer on the upper chamber of transwell system (Corning) and measured by TEER using the Millicell Electrical Resistance System (ERS)-2 (EMD Millipore, Billerica, United States). Then fresh neutrophils were added into the upper chambers and *E. coli* in the bottom chambers as illustrated in Figure 1E. All cells were cultured in a DMEM medium (Gibico) supplemented with 10% FBS at 37°C in a 5% CO_2_ atmosphere. Images of RIMVECs and neutrophils were captured by a confocal microscopy (Leica, SP8). Then *E. coli* were infected with transendothelial neutrophils at the bottom chambers of transwell for 4 h. IL-1α or LPS were added in the co-culture medium at the final concentration of 1 μg/mL or 10 ng/mL, respectively. Lastly, both the extracellular and intracellular *E. coli* (neutrophils, 5 × 10^8^ cells) were collected to calculate using the colony count technique (CFUs) according to previous study (Liu et al., 2016).

### Western Blot

RIMVECs were collected from the transwell system. RIMVECs were lysed in 1 mL of RIPA lysis buffer with 10 μL phenylmethanesulfonyl fluoride (PMSF, 1 mmol/L, Beyotime) on the ice for 20 min. Then the cell lysates were used to gain the whole proteins by centrifugation at 15,000 rpm for 12 min and proteins were quantified by the BCA method (Pierce). Fifty microgram of proteins were used to detect the expression of IL-1α by Western Blot assay. Briefly, separation of proteins used SDS-PAGE with 15% polyacrylamide gels and transferred onto a PVDF membranes (Beyotime). The primary antibody of rabbit anti- IL-1α (a dilution of 1: 1000, Invitrogen) was incubated with membranes at 4°C overnight and secondary antibody of goat anti-rabbit antibody (a dilution of 1: 3000, Beyotime) covered the membranes for 1 h at room temperature. Gray values of protein bands were quantified by ImageJ software.

### LPS Detection

Concentrations of LPS were determined by a LAL (Limulus Amebocyte Lysate, LONZA) assay. Briefly, the samples including LPS were diluted in the free endotoxin water and detected by LAL reagent (sensitivity 0.125 EU/mL) as previous published protocols (Mitra et al., 2014). 2.5 EU of LPS approximated 1 ng.

### Immunostaining and Confocal Microscopy

RIMVECs were seeded on glass coverslips (15 mm, NEST) in a 24-well plate and incubated with LPS for 4 h. Then cells were fixed by 4% paraformaldehyde and incubated with the primary antibodies, rabbit anti-IL-1α (a dilution of 1: 500, Invitrogen) at 4°C overnight. After PBS washed twice, cells were incubated with the FITC-labeled goat anti-rabbit IgG (H + L) (a dilution of 1: 1000, Beyotime) at 4°C for 2 h, which was used to visualize the IL-1α protein. Images were captured by the LAS AF Lite software (Leica).

### Protein Preparation and iTRAQ Labeling

RIMVECs were treated with IL-1α (final concentration of 10 ng/mL) at 37°C incubated in a 5% CO_2_ atmosphere for 0, 2, 4, and 8 h. The proteins of RIMVCEs were extracted by RIPA lysate buffer (Beyotime) and quantified by a BSA kit (Beyotime). The concentrations of sample proteins were detailed in Supplementary Table S1. Two hundred microgram proteins were incubated in iTRAQ-4-plex kit (AB Sciex, PN: 4352135) proteolysis. The iTRAQ labeled, LC-MS/MS analysis and MALDI-TOF-TOF identifications were conducted by BIOMS company. Labels of 114, 115, 116, and 117 are represented 2, 4, 6, and 0 h treatment, respectively (Supplementary Table S1).

### LC-MS/MS Analysis Based on TripleTOF^TM^ 5600

The iTRAQ labeled samples were run though Durashell-C18 column (4.6 mm × 250 mm, 5 μm 100 AÅ, Agela, Catalog Number: DC952505-0) and dissolved in mobile phase A, which was 2% acetonitrile in water and phase B was 98% acetonitrile in water. The gradient elution program was: 0–5 min, 5% B; 5–35 min, 8% B; 35–62 min, 32% B; 62–64 min, 95% B; 64–68 min, 95% B; 68–72 min, 5% B. The injection volume was 3 μL and the flow rate was 0.7 mL/min. The parameters of mass spectrometry were: Ion spray voltage:2.3 kv; GS1:4; Curtain gas:35; DP:100; Top MS, m/z:350-1250; accumulation time: 0.25 s; product ion scan: IDA mumber:30; m/z:100-1500; accumulation time:0.1 s; Dynamic exclusion time: 25 s; Rolling CE: enabled; Adjust CE when using iTRAQ reagent: enabled; CES:5. Analysis of iTRAQ mass spectrometry by TripleTOF^TM^ 5600 system using the software of ProteinPilot 4.0 (AB Sciex) and the database come from http://www.uniprot.org.

### Data Availability

iTRAQ-based quantitative mass spectrometry proteomics data had been deposited to the ProteomeXchange Consortium via the PRIDE (Perez-Riverol et al., 2019) partner repository with the dataset identifier PXD019561.

### Statistical Analysis

The significant differences between two groups were calculated using unpaired *t*-test with between two groups or one-way ANOVA among multiple groups and performed by GraphPad Prism 8.0 software. Results were expressed as means ± SD. Values are represented as column diagram (^∗^*P* < 0.05; ^∗∗^*P* < 0.01; ^∗∗∗^*P* < 0.001). All animals were used to analyze including both live and dead rats.

## Results

### IL-1α Prevented *E. coli* Infection *in vivo* and *in vitro*

To investigate whether IL-1α can impact on the innate immunity against bacterial infection, we firstly built the *in vivo* model of *E. coli* infected rats (Figure 1A) and the infection on co-culture system of neutrophils and endothelial cells *in vitro* (Figure 1E). We found that LPS of *E. coli* promoted the release of TNF-α, IL-6, and IL-1β in the serums of infected rats, while LPS suppressed IL-1α, IL-8, and ICAM-1 (Figure 1B). It suggested that LPS might damage endothelial-neutrophil interaction due to interrupt the inflammation. Since IL-1α can secrete from endothelial cells as well as the IL-8 and ICAM-1 are crucial for neutrophils recruitment (Gunther et al., 2017). Therefore, based on our previous study as well (Liu et al., 2016), we hypothesized that IL-1α acted as the important role on endothelial cells to activate the innate immunity. Next, results also confirmed IL-1α could increase the survival of LPS induced the *E. coli* infected rats (Figure 1C) and decrease the bacterial loading in rat colon (Figure 1D), suggesting that IL-1α could prevent the LPS induced bacterial expansion *in vivo*. For more clearing illustrated the function of IL-1α on endothelial-neutrophil interaction, we employed a Transwell system to co-culture of rat intestinal microvascular endothelial cells (RIMVECs) and neutrophils to evaluate the bacterial killing ability of transendothelial neutrophils. As Figure 1F showed, IL-1α treatment decreased both the intracellular and extracellular *E. coli*, suggesting that the bacterial killing activity of transendothelial neutrophils was enhanced by IL-1α.

### Endothelial-Derived IL-1α Was Inhibited by LPS Though Damaging Endothelial Cells and Neutrophils

**FIGURE 2:**
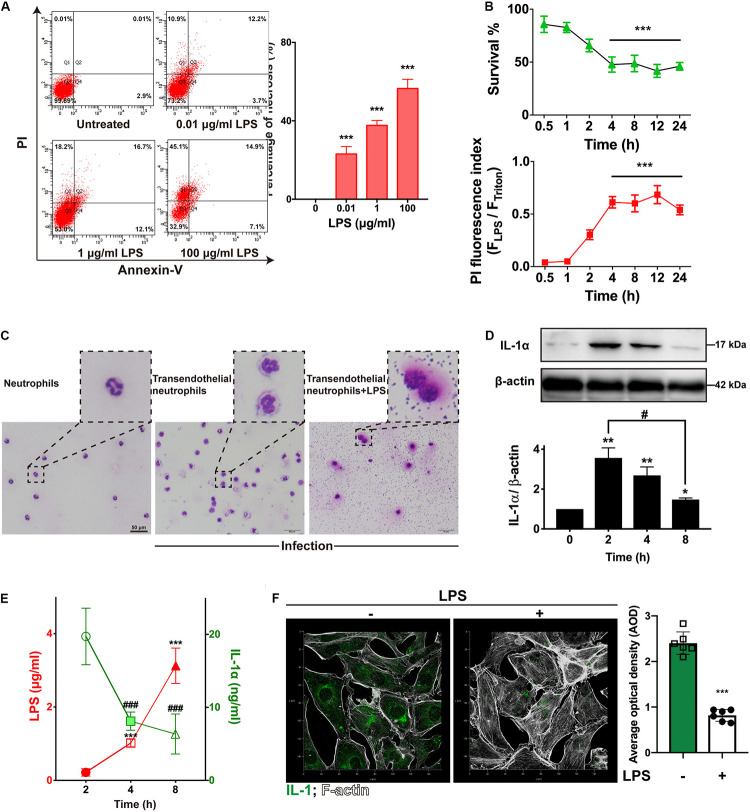
LPS damaged endothelial cells and neutrophils to suppress IL-1α. **(A)** LPS induced RIMVECs necrosis. Different concentrations of LPS (0–100 μg/mL) were treated RIMVECs for 4 h. Then cells were labeled by PI and Annexin-V staining. Apoptosis and necrosis of RIMVECs (at least 10,000 cells) were distinguished by flow cytometry assay. The percentage of Q1 and Q2 area were counted from A presented the necrotic RIMVECs. **(B)** LPS induced cytotoxicity of RIMVECs in a time dependent manner. LPS (1 μg/mL) treated RIMVECs for different time points (0.5–24 h). Then the survival of RIMVECs were determined by WST-1 assay. PI positive RIMVECs were recorded by a plate reader (SpectraMax M5) at the excitation wavelength of 535 nm and emission wavelength of 615 nm. The detached RIMVECs were also counted. **(C)** Transwell system were treated with 1 μg/mL LPS for 4 h. Transendothelial neutrophils were dyed with Switzerland staining. Images were captured by an optical microscope (Olympus). **(D)** The expression of IL-1α in co-culture system was decreased in a time dependent manner. **(E)** LPS suppressed the release of IL-1α. IL-1α release and concentrations of LPS were detected by ELISA or LAL (Limulus Amebocyte Lysate) assay, respectively. **(F)** The immunofluorescence of IL-1α in RIMVECs with or without LPS treatment. Values represent the mean ± SD (*P < 0.05; **P < 0.01; ***P < 0.001; ^###^*P* < 0.0001, ^#^*P* < 0.05, *n* = 6 or three independent biological repetition).

LPS from *E. coli* are frequently reported that impairs endothelial cells (Yang et al., 2016; Zhou et al., 2018; Huang et al., 2019). To investigate how LPS impacted RIMVECs, we utilized a double staining of PI and Annexin-V to sort the LPS treated RIMVECs by flow cytometry. As showed in Figure 2A, LPS induced RIMVECs necrosis in a dose dependent manner (4 h treatment) and further led cell death and impaired cellular membranes in a time dependent way (Figure 2B). It was consisted with previous reports that LPS injury tissue and led cell necroptosis (Li et al., 2016; Huang et al., 2019). In fact, LPS action on endothelial cells is intend to occur an inflammation within endothelial-neutrophil interactions (Al-Banna et al., 2013; Shi et al., 2014), and IL-1 family is closely related with inflammation (Dinarello, 2011; Liu et al., 2016; Gunther et al., 2017). In addition, we found that LPS damaged the transendothelial neutrophils and led the bacterial escape from cells (Figure 1C). Take it together, IL-1α prevented *E. coli* infection by enhancing the killing ability of transendothelial neutrophils, while LPS destroyed it. Therefore, we next detected the amount of LPS and IL-1α in the co-culture system to reveal the function of each other. We found that the expression of endothelial-derive IL-1α was inhibited by LPS (Figure 1D). Results also indicated that the release of LPS and IL-1α in co-culture transwell system was competitive (Figure 1E) and the addition of LPS did weaken the expression of IL-1α (Figure 1F). Altogether, IL-1α might be the key factor that impact bacterial killing during endothelial-neutrophil interaction.

### IL-1α Facilitated Neutrophil Killing via Sustaining Oxidative Phosphorylation Activity

**FIGURE 3:**
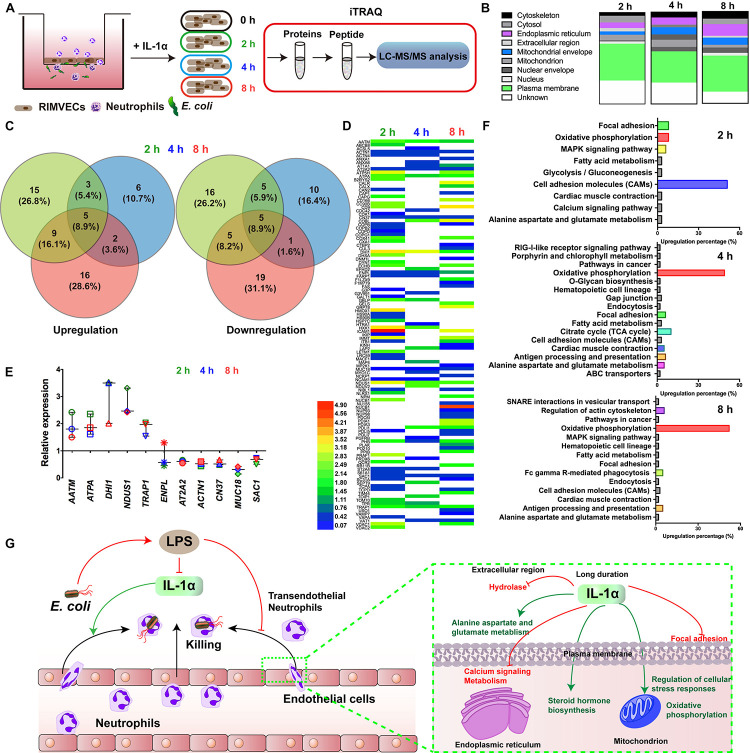
Oxidative phosphorylation was required for IL-1α activated-endothelial cells. **(A)** Scheme of the iTRAQ experimental set-ups. **(B)** The regulated proteins were mostly distributed on plasma membrane. RIMVECs were treated with IL-1α (10 ng/mL) for 2, 4, or 8 h and the proteins regulation of RIMVECs were analyzed by iTRAQ. **(C)** Venn diagram showed the percentage of upregulated and downregulated proteins, which were drawn by Venny 2.1.0 software online. **(D)** Heat map of the differential protein analyzed by iTRAQ. RIMVECs were treated with IL-1α (10 ng/mL) for 2, 4, or 8 h. At every time points, the proteins of RIMVECs were collected for iTRAQ assay. The image of heat map was made by Heml software. **(E)** The common expressed proteins of RIMVECs induced by IL-1α at all the treated times (*n* = 3, down-up regulated difference >1.5-fold change). **(F)** IL-1α upregulated the cell adhesion molecules (CAMs) of RIMVECs at 2 h treatment, while mainly triggers oxidative phosphorylation at 4 and 8 h. **(G)** Scheme of endothelial IL-1α facilitated transendothelial neutrophil killing.

For deeply exploring the modulatory function of IL-1α, iTRAQ labeling technology combined with mass spectrometry proteomic analysis were used for investigating the differentially expressed proteins of RIMVECs from Transwell system (Figure 3A). We first analyzed the location of different proteins and found that most of them were disturbed on plasma membrane compared to 0 h (2 h about 33%, 4 h was 35% and 8 h was 40%, Figure 3B). Venn diagram showed that there had 31 proteins of upregulation and 29 proteins of downregulation at 2 h, for 4 h treatment, 14 upregulations, 19 downregulations and 31 upregulations, 26 downregulations at 8 h (Figure 3C). As showed in the heat map (Figure 2D), compared to untreated group, there are 63, 37, or 63 proteins significant regulation in 2, 4, or 8 h treatment, respectively (Supplementary Table S2). These data showed that IL-1α is a complex regulation in protein levels. Combating with our previous results that IL-1α enhanced both extracellular and intracellular bacterial killing of transendothelial neutrophils mainly focused on the long-time treatment (4–8 h, Figure 1F). Thus, we believe that the common regulated proteins in all time points is playing the key role. Therefore, we analyzed that 11 proteins were sustained to regulate significantly, among them 5 proteins upregulation and 5 proteins downregulation (Figure 3E). Interesting, one protein named Endoplasmin (ENPL) was upregulated at 2 h while downregulated at 4 and 8 h. All the information and analysis of the common regulated proteins were detailed in Table 1. Nevertheless, the function and location of ENPL still remained unclear. But it was worth noting that there were two proteins including ATPA and NDUS1 upregulated responded to oxidative phosphorylation.

**TABLE 1:** Functional analysis of common regulated proteins.

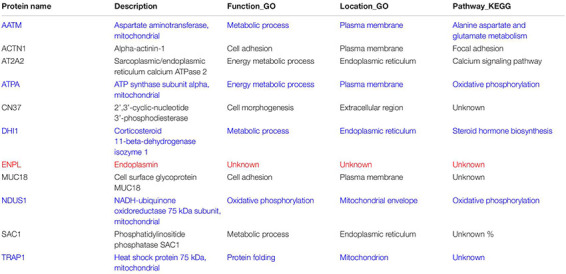

**The blue marked table frames represented the upregulated proteins, while unmarked were the downregulated proteins. Red marked the protein had both upregulation and downregulation.**

Next, we deduced that oxidative phosphorylation was required for IL-1α-dependent activation of endothelial cells. Therefore, in order to more detail the functional regulation, we analyzed that the pathway cascade in 2, 4, and 8 h. As showed in Figure 3F, cell adhesion molecules (CAMs) major responded to 2 h treatment of IL-1α. It was consisted with our previous results that LPS targeted ICAM-1 and IL-1α to affect the bacterial killing of transendothelial neutrophils. As we proposed that the accumulation of IL-1α is the importance factor that activates the transendothelial neutrophils, we found that in long time treatment of IL-1α, oxidative phosphorylation is mainly regulated pathways (4 h was 49% and 8 h was 52%). Oxidative phosphorylation is vital for physiological function regulations in most eukaryocyte, especially in endothelial cells (Papa et al., 2012; Montorfano et al., 2014; Nath and Villadsen, 2015). We further found that the oxidative phosphorylation induced by IL-1α occurred on mitochondrial envelope (Table 1). Take it together, it revealed that additional IL-1α treated in the *E. coli* infected co-culture system of endothelial cells and neutrophils was originally enhanced the adhesion of neutrophils and then promoted the migration of neutrophils by triggering oxidative phosphorylation to generates ROS. It consisted with the fact that neutrophil transmigration across endothelial cells was essential for ROS and sustaining the inflammatory response (Mittal et al., 2017). Combining with these researches, our data further revealed a new function that IL-1α induced the oxidative phosphorylation of endothelial cells to assist transendothelial neutrophils killing.

## Discussion

Endothelial cells not only form the physical barrier against pathogenic invasion (Sturtzel, 2017; He et al., 2020), but also have the regulation of transendothelial neutrophils. Indeed, endothelial cells turn into the activated form during the migration of neutrophils (Mittal et al., 2017; Folco et al., 2018). The action of transendothelial migration is a series of complex physical and biological processes (Kolaczkowska and Kubes, 2013; Mooren et al., 2014). In general, bacterial infection is connected with endothelial cell activation and neutrophil migration. Many bacterial pathogens utilize their toxins to disrupt endothelial integrity and inhibited immune cells as confirmed as our results (Figure 2) and further trigger the inflammation (Kim et al., 2010; Amedei and Morbidelli, 2019; Le Guennec et al., 2020). However, we do not know the role of endothelial cells act on the transendothelial migration during innate immunity. In this study, we focused on endothelial-derived IL-1α, because accumulation of IL-1α release from endothelial cells is the signaling for transendothelial migration of neutrophils (Burzynski et al., 2015). Our previous results also showed that IL-1α activated endothelial cells to promote neutrophil killing by improving the lysozyme activation (Liu et al., 2016). In this paper, we also found that IL-1α promoted bacterial killing of transendothelial neutrophils (Figure 1F). More interesting, LPS inhibited the release of IL-1α *in vivo* and *in vitro* (Figures 1, 2). It indicated that LPS damaged endothelial cells and interrupted the bacterial killing of transendothelial neutrophils was connected with IL-1α. Unlike other researches show that IL-1α has the ability to induce neutrophil-endothelial cell adhesion (Macmillan et al., 2010), these findings performed a novel link of IL-1α on the bacterial killing activity within transendothelial migration.

IL-1α modulates both endothelial cells and neutrophils, involving inflammation (Dinarello, 2011; Burzynski et al., 2015; Altmeier et al., 2016). Endothelial cells are the center role involved in neutrophils and inflammation (Sturtzel, 2017; Rohlenova et al., 2018). Therefore, we targeted on the different proteins of endothelial cells by IL-1α treatment. It worth to note that IL-1α have a specific time dependent function on protein regulations of endothelial cells. In the early state, IL-1α might promote neutrophil recruitment by upregulating ICAM, while oxidative phosphorylation was continuously required in the later stages (Figure 3F). These results had the important significance, it well explained that the generation of ROS was continuously required during endothelial-neutrophil interaction and inflammation (Papa et al., 2012; Montorfano et al., 2014; Mittal et al., 2017; Xu et al., 2019). More importantly, we also selected for specific functional proteins in detail (Figure 3D and Table 1), we believed these proteins would useful for further study of IL-1α on bactericidal activity of transendothelial neutrophils.

## Conclusion

We demonstrated that endothelial-derived IL-1α was critical for neutrophil killing during endothelial-neutrophil interaction. As illustrated in Figure 3G, bacterial LPS inhibited the release of IL-1α from endothelial cells and further prevented the bacterial killing ability of transendothelial neutrophils. In turn, IL-1α was utilized as the signaling to trigger the transendothelial neutrophils killing. iTRAQ-based quantitative mass spectrometry proteomic analysis illustrated that IL-1α inhibited the hydrolase in the extracellular region, focal adhesion of plasma membrane and calcium signaling metabolism on endoplasmic reticulum. IL-1α promoted alanine aspartate and glutamate metabolism on plasma membrane, enhanced steroid hormone biosynthesis on endoplasmic reticulum and also increased the regulation of cellular stress responses and oxidative phosphorylation on mitochondrion.

## Data Availability Statement

The datasets presented in this study can be found in online repositories. The names of the repository/repositories and accession number(s) can be found in the article/ Supplementary Material.

## Ethics Statement

The animal study was reviewed and approved by the experimental protocols involving rats were gained an approval (SCXK, 2016-006). All animals were approved by the Genentech Institutional Animal Care and Use Committee at the China Agricultural University (SYXK, 2016-0008).

## Author Contributions

HD and GH conceived the project. XL, XM, and HD did the research design. XL, HZ, and SH performed the experiments. XL, GH, and HD did the data analysis and wrote the manuscript. All authors contributed to the article and approved the submitted version.

## Conflict of Interest

The authors declare that the research was conducted in the absence of any commercial or financial relationships that could be construed as a potential conflict of interest.

## Abbreviations

*E. coli*: *Escherichia coli*
IL-1 α: Interleukin-1 α
LPS: Lipopolysaccharide
RIMVECs: rat intestinal mucosa microvascular endothelial cells
iTRAQ: isobaric tags for relative and absolute quantification.
