# Supplementary material for: Endothelial-Derived Interleukin-1α Activates Innate Immunity by Promoting the Bactericidal Activity of Transendothelial Neutrophils
Source: Front Cell Dev Biol. 2020 Jul 7;8:590. doi: 10.3389/fcell.2020.00590 (PMC7358461; doi:10.3389/fcell.2020.00590)
Supplement: Supplementary file 1 [file Data_Sheet_1.docx]

**Supplement information for**

**Endothelial interleukin-1alpha activates innate immunity by promoting the bactericidal activity of transendothelial neutrophils**

Xiaoye Liu ^1,2,3^, Hui Zhang ^2^, Shangwen He^2^, Xiang Mu^2^, Ge Hu^2,^ *, Hong Dong ^2,^ *

1. Beijing Traditional Chinese Veterinary Engineering Center and Beijing Key Laboratory of Traditional Chinese Veterinary Medicine, Beijing University of Agriculture, No.7 Beinong Road, Changping, Beijing 102206, China.

2. Department of Mechanics and Engineering Science, College of Engineering, Academy for Advanced Interdisciplinary Studies, and Beijing Advanced Innovation Center for Engineering Science and Emerging Technology, College of Engineering, Peking University, No.5 Yiheyuan Road, Haidian, Beijing 100871, China

3. Beijing Advanced Innovation Center for Food Nutrition and Human Health, College of Veterinary Medicine, China Agricultural University, No.2 Yuanmingyuan West Road, Beijing 100193, China

Corresponding author: *Dong Hong, email: [donghongbua@163.com](mailto:donghongbua@163.com). *Ge Hu, email: [huge@bua.edu.cn](mailto:huge@bua.edu.cn).

**Tables**

**Table S1. Concentrations of proteins from RIMVECs prepared for iTRAQ. Related to Figure 2.**

| **Samples** | **2 h** | **4 h** | **8 h** | **0 h** |
| --- | --- | --- | --- | --- |
| **iTRAQ labels** | 114 | 115 | 116 | 117 |
| **Concentrations (mg/mL)** | 0.29 | 0.60 | 0.24 | 0.77 |

**Table S2. The differential proteins analyzed by iTRAQ. Related to Figure 2C.**

| No. | **2 h** | |  | **4 h** | |  |  | **8 h** | |
| --- | --- | --- | --- | --- | --- | --- | --- | --- | --- |
|  | Name | Ratio vs 0 h |  | Name | Ratio vs 0 h |  |  | Name | Ratio vs 0 h |
| 1 | AATM | 2.421029091 |  | AATM | 1.803017974 |  |  | AATM | 1.499685049 |
| 2 | ABCB8 | 1.55596602 |  | ACSL5 | 0.346736789 |  |  | ACTN1 | 0.602559626 |
| 3 | ACTN1 | 0.436515808 |  | ACTN1 | 0.524807513 |  |  | ANXA1 | 0.630957425 |
| 4 | ACTN4 | 0.685488224 |  | ACTN4 | 0.704693079 |  |  | AT1A1 | 1.445440054 |
| 5 | AT2A2 | 0.654636085 |  | ANXA6 | 0.608134985 |  |  | AT2A2 | 0.544502676 |
| 6 | ATP5H | 3.250873089 |  | AT1A1 | 0.510505021 |  |  | ATP5H | 2.728977919 |
| 7 | ATPA | 2.355048895 |  | AT2A2 | 0.608134985 |  |  | ATPA | 1.853531957 |
| 8 | B2RYS2 | 2.269865036 |  | ATPA | 1.614359021 |  |  | CALR | 3.28095293 |
| 9 | CAN2 | 0.4487454 |  | CDC42 | 0.597035289 |  |  | CALX | 1.706081986 |
| 10 | CAPG | 2.013724089 |  | CN37 | 0.515228629 |  |  | CAP1 | 0.428548515 |
| 11 | CC90B | 2.558585882 |  | COBL | 2.187762022 |  |  | CD166 | 2.992264986 |
| 12 | CD9 | 2.53512907 |  | DHI1 | 3.499452114 |  |  | CD9 | 3.698282003 |
| 13 | CDK1 | 0.291071713 |  | EFHD2 | 1.629295945 |  |  | CN37 | 0.666806817 |
| 14 | CN37 | 0.487528503 |  | ENPL | 0.57543987 |  |  | COBL | 3.372873068 |
| 15 | COPB | 0.586138189 |  | FARP1 | 0.457088202 |  |  | COPB | 0.337287307 |
| 16 | COPB2 | 0.519995987 |  | G3V991 | 0.636795521 |  |  | COX41 | 2.333457947 |
| 17 | COPD | 0.40926069 |  | GBLP | 1.67494297 |  |  | CTBP2 | 0.072443597 |
| 18 | COPG1 | 0.691830993 |  | HTRA1 | 0.1127197 |  |  | CUL3 | 0.549540877 |
| 19 | COX41 | 2.333457947 |  | HXK1 | 2.355048895 |  |  | DHI1 | 2.013724089 |
| 20 | CXA1 | 2.032356977 |  | LAP2 | 1.499685049 |  |  | DNM1L | 0.363078088 |
| 21 | DHI1 | 3.53183198 |  | MAP4 | 1.406048059 |  |  | ECHB | 2.147830009 |
| 22 | DHSA | 2.558585882 |  | MUC18 | 0.310455889 |  |  | ENPL | 1.29419601 |
| 23 | DYN1 | 1.976969957 |  | MYO1C | 0.597035289 |  |  | F1LZX9 | 1.819700956 |
| 24 | ECHB | 2.187762022 |  | NCAM1 | 0.316227794 |  |  | F1M779 | 0.334194988 |
| 25 | ENPL | 0.452897608 |  | NDUS1 | 2.466038942 |  |  | FAS | 0.457088202 |
| 26 | FARP1 | 0.544502676 |  | NDUV2 | 2.147830009 |  |  | GELS | 2.466038942 |
| 27 | G3P | 0.380189389 |  | PDLI1 | 1.995262027 |  |  | GRP78 | 3.311311007 |
| 28 | GALT1 | 0.322106898 |  | PGFRB | 0.285759091 |  |  | ICAM1 | 3.311311007 |
| 29 | GBLP | 2.443430901 |  | PRDX1 | 0.424619585 |  |  | IMMT | 2.937649965 |
| 30 | HMOX1 | 3.767038107 |  | PRDX6 | 0.237683997 |  |  | ITB1 | 1.629295945 |
| 31 | HS90A | 0.549540877 |  | S12A4 | 0.55975759 |  |  | K2C1 | 13.18257046 |
| 32 | HS90B | 0.549540877 |  | SAC1 | 0.724435985 |  |  | KINH | 0.401790798 |
| 33 | HSP7C | 1.570363045 |  | SEPT9 | 1.737800956 |  |  | LAP2 | 0.322106898 |
| 34 | HXK1 | 3.732501984 |  | TOIP1 | 1.599557996 |  |  | MP2K1 | 0.398107201 |
| 35 | ICAM1 | 5.248075008 |  | TPR | 1.432188034 |  |  | MUC18 | 0.416869402 |
| 36 | IKIP | 0.337287307 |  | TRAP1 | 1.541700006 |  |  | NCKP1 | 0.602559626 |
| 37 | IMMT | 3.34194994 |  | VAT1 | 0.478630096 |  |  | NDUS1 | 2.421029091 |
| 38 | ITB1 | 0.636795521 |  |  |  |  |  | NIBL1 | 0.704693079 |
| 39 | LETM1 | 2.398833036 |  |  |  |  |  | NPM | 1.853531957 |
| 40 | LRC59 | 0.432513803 |  |  |  |  |  | NU155 | 0.539510608 |
| 41 | MACF1 | 0.679203629 |  |  |  |  |  | NUCB1 | 4.613175869 |
| 42 | MUC18 | 0.147231296 |  |  |  |  |  | NUP93 | 0.405508488 |
| 43 | NCAM1 | 0.134276494 |  |  |  |  |  | NUP98 | 0.57543987 |
| 44 | NDUS1 | 3.311311007 |  |  |  |  |  | PDC6I | 0.492039502 |
| 45 | NIBL1 | 0.672976673 |  |  |  |  |  | PDIA1 | 3.133285999 |
| 46 | NLRX1 | 1.499685049 |  |  |  |  |  | PDIA3 | 2.964831114 |
| 47 | NUCB1 | 2.654606104 |  |  |  |  |  | PDLI1 | 1.976969957 |
| 48 | PDLI5 | 0.405508488 |  |  |  |  |  | PDLI5 | 0.685488224 |
| 49 | PHB | 2.032356977 |  |  |  |  |  | PDLI7 | 0.636795521 |
| 50 | PRAF1 | 3.162277937 |  |  |  |  |  | PHB | 1.976969957 |
| 51 | QCR2 | 1.853531957 |  |  |  |  |  | PLAK | 1.445440054 |
| 52 | S12A4 | 0.416869402 |  |  |  |  |  | PO210 | 0.461317599 |
| 53 | S61A1 | 0.428548515 |  |  |  |  |  | PPIB | 1.958845019 |
| 54 | SAC1 | 0.505824685 |  |  |  |  |  | RB11B | 1.853531957 |
| 55 | SC31A | 0.602559626 |  |  |  |  |  | SAC1 | 0.679203629 |
| 56 | TCPA | 0.510505021 |  |  |  |  |  | SC31A | 0.334194988 |
| 57 | TCPD | 0.461317599 |  |  |  |  |  | TCPD | 0.501187205 |
| 58 | TOM70 | 1.541700006 |  |  |  |  |  | TIM44 | 1.803017974 |
| 59 | TPR | 1.570363045 |  |  |  |  |  | TRAP1 | 1.976969957 |
| 60 | TRAP1 | 2.032356977 |  |  |  |  |  | USO1 | 0.586138189 |
| 61 | VAPA | 0.474242002 |  |  |  |  |  | VAMP7 | 0.216770396 |
| 62 | VDAC1 | 2.85758996 |  |  |  |  |  | VAT1 | 0.544502676 |
| 63 | VDAC2 | 2.187762022 |  |  |  |  |  | VDAC1 | 1.819700956 |
